# Extended-view totally extraperitoneal repair for ventral hernias: a retrospective analysis of perioperative outcomes and the role of ASA score

**DOI:** 10.1007/s00423-025-03914-2

**Published:** 2025-11-26

**Authors:** Imke Emma Hannig, Nader El-Sourani, Maximilian Bockhorn, Asem Al-Salemi, Fadl Alfarawan

**Affiliations:** 1 Fakultät für Gesundheitswissenschaften, Carl von Ossietzky Universität Oldenburg, Ammerländer Heerstraße 114-118, 26129 Oldenburg, Germany; 2 Department for General - and Visceral Surgery, University Hospital Oldenburg, Klinikum Oldenburg AöR, Ra-hel-Strauss-Straße 10, 26133 Oldenburg, Germany; 3 Department for General-, Visceral- and Transplant Surgery, University Hospital Münster, Waldeyerstraße 1, 48149 Münster, Germany

**Keywords:** Asa, Complications, Etep, Length of hospital stay, Ventral hernia

## Abstract

**Purpose:**

Ventral hernias are frequently encountered in general surgery. In recent years, minimally invasive techniques, including the Extended View Totally Extraperitoneal Repair (eTEP), have gained popularity. Meta-analyses suggest that eTEP may offer both intraoperative and postoperative advantages over alternative approaches. This retrospective study aimed to evaluate intra- and postoperative complications, length of hospital stay, and the potential association between American Society of Anesthesiologists (ASA) Physical Status Classification and complication rates in patients who underwent eTEP. The findings may inform preoperative risk stratification and surgical planning based on ASA score.

**Methods:**

A monocentric, retrospective study of 95 patients who underwent eTEP for ventral hernia repair between January 2019 and December 2021 was conducted. Descriptive statistics and binary logistic regression analyses were performed to explore the association between the ASA score and perioperative complications.

**Results:**

Intraoperative complications occurred in 2.1% (*n* = 2), and postoperative complications were observed in 7.4% (*n* = 7). The median length of hospital stay was three days (IQR = 1). Logistic regression analysis did not identify a statistically significant association between the ASA score and the occurrence of either intra- or postoperative complications. Nonetheless, all observed complications arose in patients with ASA scores of ≥ II.

**Conclusion:**

eTEP repair was associated with a low complication rate and short hospitalization. While ASA classification was not a statistically significant predictor of perioperative complications in this cohort, the absence of complications in ASA I patients suggests potential relevance. These findings highlight the need for larger, prospective studies to further evaluate the role of ASA classification in risk assessment for eTEP procedures.

## Introduction

Ventral hernias constitute a substantial proportion of cases encountered in general and visceral surgery. The incidence of ventral incisional hernias has been reported to range from 2% to 20%, depending on patient-related risk factors and surgical technique [1, 2]. According to a meta-analysis of Australian population data from 2000 to 2020, the cumulative, population-adjusted incidence of ventral hernias was 20–30%. Incidence rates varied by hernia subtype, with umbilical hernias being the most common, followed by incisional hernia [3, 4]. Over time, surgical treatment strategies have undergone considerable evolution. Traditionally, open surgical repair was the standard approach, often associated with significant morbidity and prolonged recovery periods [5]. The advent of minimally invasive techniques marked a paradigm shift, with robotic-assisted surgery being a particularly notable advancement. One such minimally invasive method is the Extended View Totally Extraperitoneal Repair (eTEP), introduced in 2012 by Jorge Daes for the treatment of complex inguinal hernias [6]. Meta-analyses suggest that eTEP offers several intraoperative and postoperative advantages over conventional techniques, such as the Intraperitoneal Onlay Mesh (IPOM) approach, particularly with respect to patient outcomes [7–9]. Since the description of the eTEP approach, the published literature remains limited to predominantly single-center, retrospective series ranging from 29 to 171 patients. With 95 patients, our series represents the third-largest eTEP cohort reported to date, exceeding the sample sizes of multiple published studies including Taşdelen (*n* = 30), Bui et al. (*n* = 29), Quezada et al. (*n* = 55), and Wang et al. (*n* = 55) [10–13].

Complications associated with ventral hernia repair are well documented and can be broadly categorized into intraoperative and postoperative events. Intraoperatively, the risk of organ injury and bleeding remains a major concern, potentially leading to anesthetic complications [14]. Postoperative challenges of clinical relevance include pain, impaired mobility, hematoma, infection, ileus, seroma formation, and hernia recurrence [15–23]. A 2022 meta-analysis reported an intraoperative complication rate of 2% for the eTEP technique [16]. Surgical site infections occurred in less than 1% of cases, surgical site occurrences in 5%, Clavien-Dindo grade III-IV complications in 1%, and recurrence rates were approximately 1% [4].

Previous studies indicate that the ASA (American Society of Anesthesiologists) score correlates with perioperative risk, but its predictive value in eTEP procedures remains unclear [24–27]. The ASA score is an internationally recognized system for classifying a patient’s preoperative physical status, serving as a surrogate marker of overall morbidity. It is primarily used to estimate perioperative risk and the likelihood of postoperative complications. The classification consists of six ascending categories, each reflecting increasing levels of systemic disease and operative risk.

Studies have demonstrated that a higher ASA score is generally associated with increased intraoperative blood loss, a higher rate of postoperative complications, prolonged intensive Care Unit (ICU) stays, and elevated mortality. A study published in the British Journal of Anaesthesia reported that patients with an ASA score of IV had a 4.2-fold significantly increased risk of postoperative complications, while those with an ASA score of III had a 2.2-fold significantly increased risk [24]. Further investigations, but not involving eTEP procedures specifically, have explored this association in the context of ventral hernia repair. Lindmark et al. (2018) observed significantly higher complication rates among patients with an ASA score of III. However, this association was only evident in univariate analysis [25]. Similarly, Liang et al. (2015) identified an increased incidence of surgical site infections (SSI) in patients with an ASA score of III or above [26].

As these findings have not yet been specifically examined in the context of eTEP procedures, it is particularly relevant to investigate the relationship between ASA classification and clinical outcomes in patients undergoing eTEP repair.

This study aims to analyze perioperative complications associated with eTEP repair for ventral hernias, thereby providing more precise data for patient counseling and supporting healthcare institutions in evaluating and optimizing surgical practices. The study also investigates the relationship between ASA score and perioperative complication incidence, which could inform preoperative strategies and improve postoperative management, potentially reducing both patient risk and healthcare costs.

Previous studies indicate that the ASA score correlates with perioperative risk in general surgery and specifically in some hernia repairs [25, 26, 28], including findings of higher complication rates in ASA ≥ III patients [25, 26]. However, its specific predictive value within the context of the increasingly adopted eTEP approach for ventral hernias remains unexplored. The unique anatomical dissection planes, potential for longer operative times, and specific complication profile (e.g., seroma, retromuscular bleeding) associated with eTEP warrant procedure-specific risk assessment [16, 17, 29]. As these findings have not yet been specifically examined in the context of eTEP procedures, it is particularly relevant to investigate the relationship between ASA classification and clinical outcomes in patients undergoing eTEP repair. This study aims to analyze perioperative complications associated with eTEP repair for ventral hernias, specifically evaluating the utility of the ASA score as a predictor within this distinct surgical context, thereby providing more precise data for patient counseling.

## Materials and methods

### Study population and variables

**Fig. 1:**
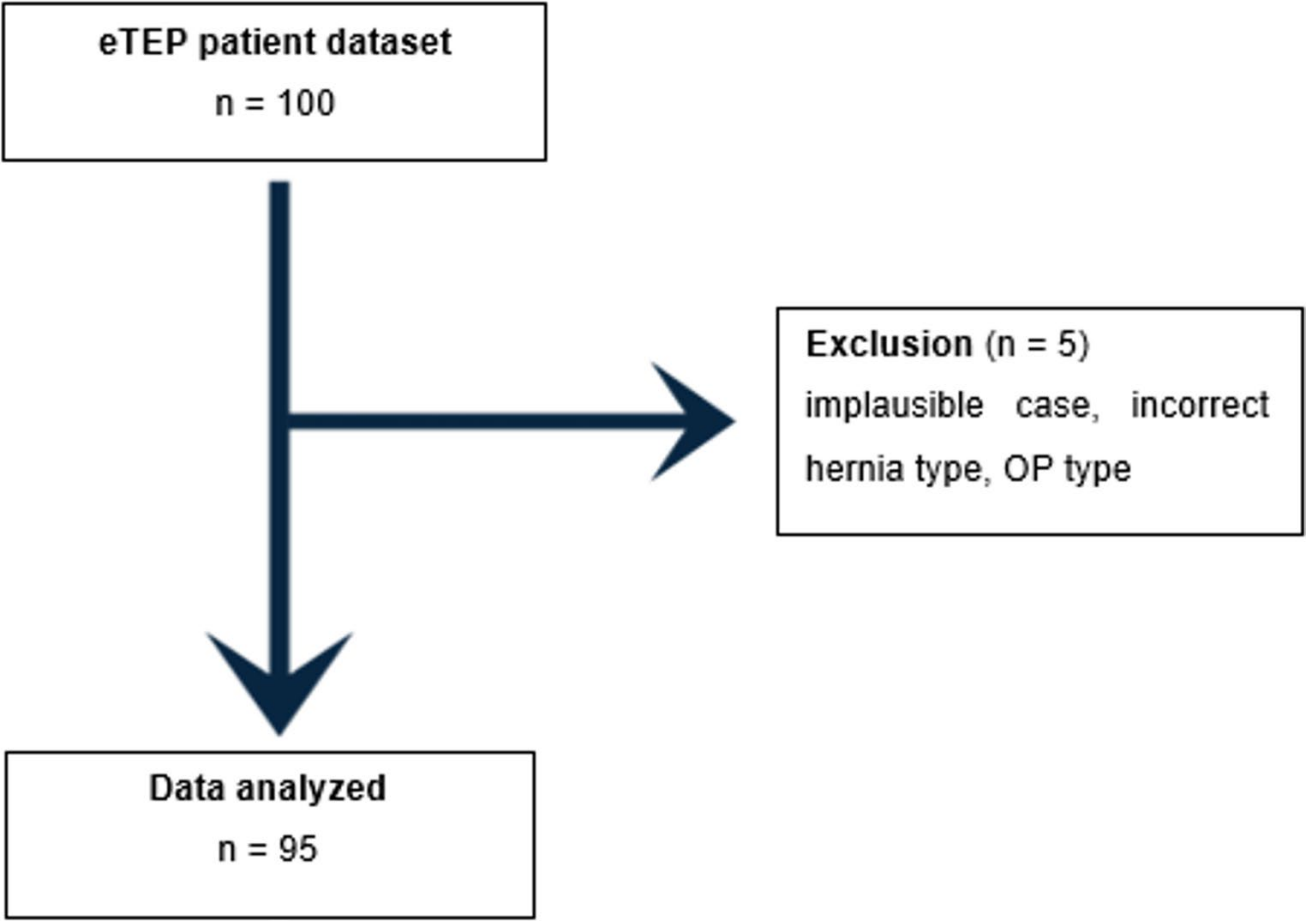
Flowchart depicting patient inclusion

This study is a single-center, retrospective cohort analysis based on a dataset derived from patient records at Klinikum Oldenburg, compiled in Microsoft Excel. The dataset was anonymized by assigning unique identification codes. It includes patients who underwent treatment in the Department of General and Visceral Surgery at Klinikum Oldenburg between January 2019 and December 2021. All surgical procedures were performed by a single surgeon. For the purposes of this analysis, only patients with ventral hernias who underwent non-robotic eTEP repair and exhibited clinically plausible parameter values, as confirmed through medical review, were included. Patients under 18 years of age and those with terminal illness were excluded. Cases involving other techniques than eTEP were excluded. Only ventral hernias were included. Lateral hernias (EHS classification “L”) were excluded to maintain a homogeneous cohort. Lateral hernias differ anatomically and biomechanically from midline (M1-M5) hernias [30–32]. This restriction was implemented to ensure consistency and clinical relevance. Rectus diastasis, when present, was repaired simultaneously during the ventral hernia procedure as part of the eTEP repair. Fascial plication of the linea alba was performed after reduction of the hernia sac and before mesh placement in the retromuscular plane. Data entries deemed implausible by at least two independent medical reviewers (physicians or senior medical students) were omitted. Based on these inclusion and exclusion criteria, 5 out of 100 patients were excluded from the final analysis (Fig. 1).

### Outcome

The primary outcomes were defined as the occurrence of any intraoperative or postoperative complications. Intraoperative complications were classified as any adverse event requiring therapeutic intervention occurring between skin incision and the patient’s transfer from the operating room. Postoperative complications were defined as any newly diagnosed condition arising after completion of the surgical procedure and during the index hospital stay, necessitating either medical or surgical treatment. Postoperative complications during hospitalization were recorded and classified according to the Clavien-Dindo grading system to ensure standardized reporting of severity. Secondary outcomes included length of hospital stay and the incidence of ventral hernia recurrence within one year following surgery, serving as measures of postoperative recovery and long-term procedural durability.

### EHS classification

In 2009, the European Hernia Society (EHS) established a classification system for ventral hernias, distinguishing between primary (e.g., umbilical, epigastric) and secondary hernias (e.g., incisional) based on defect size and anatomical location [33]. In this study, hernias were classified intraoperatively according to the EHS system, and this classification was subsequently used for analysis, ensuring improved comparability.

### Surgical technique

To elucidate the relationship between surgical technique and the occurrence of complications, the eTEP procedure for ventral hernia repair is outlined here using the example of an umbilical hernia. The procedure begins with a skin incision lateral to the xiphoid process at the level of the costal margin. The anterior rectus sheath is dissected to permit the insertion of the initial trocar into the retromuscular space. Blunt dissection of this space is then performed under endoscopic guidance. Two additional trocars are placed along the lateral aspect of the left rectus sheath to enable complete caudal dissection beyond the arcuate line. For umbilical hernias, the crossover technique is employed. This involves incising the medial edge of the left rectus sheath cranial to the umbilicus, while preserving the underlying peritoneum at the dorsal aspect of the linea alba. A preperitoneal plane is then developed by separating the preperitoneal fat and peritoneum from the linea alba. Subsequently, the posterior rectus sheath on the right side is incised lateral to the linea alba, thereby allowing access to the right retromuscular space. The hernia sac is then identified and meticulously dissected from the defect, which is closed using a continuous suture. Finally, a large-pore, non-absorbable mesh is placed in the retromuscular space without fixation [7]. The detailed surgical steps of the non-robotic eTEP approach, including representative intraoperative visuals, have been comprehensively described and illustrated in our prior publication focusing specifically on the technique and its outcomes [9, 29, 34]. As the primary aim of the present study was to analyze perioperative complications in relation to ASA score rather than introduce a novel technique, readers are referred to this previous work for in-depth technical depiction [9].

### Follow-up

Follow-up assessments were conducted at 30 days and 12 months postoperatively. The one-year follow-up was performed via a structured telephone interview conducted by medical staff using the Ventral Hernia Recurrence Inventory (VHRI). During the interview, patients were asked three standardized questions: (1) Do you experience pain or discomfort at the surgical site? (2) Is there any swelling present? (3) Do you suspect a recurrence of the hernia? If the response to any of these questions was positive, patients were invited to the surgical outpatient clinic for further clinical evaluation. This included an initial ultrasound examination, supplemented by computed tomography if indicated. Recurrence was confirmed or ruled out based on these diagnostic findings. Postoperative complications were systematically recorded for a period of 30 days following the operation.

### Statistical analysis

Data analysis was performed using descriptive statistics in Statistical Package for the Social Sciences (SPSS, version 29.0, IBM Corp., Armonk, NY, USA). Variables were initially classified based on their measurement scales, and descriptive statistics were presented in tables. The quartiles and interquartile range (IQR) were calculated based on percentiles determined using the weighted average method in the SPSS Explore module. This approach allows for precise interpolation of the quartiles. Normality of the data was assessed using the Shapiro-Wilk test and the variances were examined using the Levene test. Based on the level of measurement and distribution of the variables, appropriate statistical tests were applied. Group comparisons of means were conducted using one-way ANOVA for metric variables that were normally distributed and demonstrated homogeneity of variance. For metric variables that were not normally distributed, the Kruskal-Wallis test was used. Nominal, ordinal, and categorical variables were analyzed using the chi-square test of independence, as appropriate. To assess the association between intra- and postoperative complications and ASA classification, univariate and multivariate binomial logistic regression analyses were conducted. In these models, intraoperative and postoperative complications served as dependent variables, with the ASA score and additional covariates included as independent variables. A two-tailed significance level of 5% (*p* < 0.05) was applied to all statistical tests. A post-hoc power analysis was performed using statistical software. Incidence rates reported in the literature, along with data obtained from the present study, were used as input parameters.

## Results

### Patient demographics

There was a gender imbalance in the patient cohort, with approximately 62% (*n* = 59) male and 38% (*n* = 36) female patients. The median age was 51.88 ± 13.13 years. According to EHS classification, hernia types were distributed as follows: M1 in 2.1% (*n* = 2) of cases, M2 in 45.3% (*n* = 43), M3 in 76.8% (*n* = 73), M4 in 9.5% (*n* = 9), and M5 in 3.2% (*n* = 3). Rectus diastasis was identified in 20% (*n* = 19) of patients, and 34.7% (*n* = 33) presented with a combined hernia, defined as the presence of multiple concurrent hernia defects. The median hernia defect size was 6 cm² (IQR 3.5–13), and the median mesh size implanted was 450 cm² (IQR 300–450). The median operative time was 108 min (IQR 81–141.50.50). A detailed overview is provided in Table 1.

**Table 1:** Patient demographics

	Total	ASA score I	ASA score II	ASA score III	*p*-value
Number of patients, n (% of the patient dataset)	**95 (100)**	**6 (6.3)**	**63 (66.3)**	**26 (27.4)**	-
Gender (female/male), n (%)	36/59 (37.9/62.1)	3/3 (50/50)	26/37 (41.3/58.7)	7/19 (26.9/73.1)	0.366
Age at time of surgery, mean ± SD years	51.88 ± 13.13	45.33 ± 11.60	49.51 ± 11.95	60.23 ± 13.31	**< 0.001**
BMI, median (IQR)	31 (9)	25 (9.25)	30 (8)	35.9 (11.25)	**0.005**
Cardiovascular patients, n (%)	49 (51.6)	0 (0)	25 (39.7)	24 (92.3)	**< 0.001**
Lung disease patients, n (%)	2 (2.1)	0 (0)	1 (1.6)	1 (3.8)	0.743
Hepatic insufficiency, n (%)	0 (0)	0 (0)	0 (0)	0 (0)	-
Diabetes mellitus, n (%)	12 (12.6)	0 (0)	7 (11.1)	5 (19.2)	0.363
Intake of anticoagulation, n (%)	13 (13.7)	0 (0)	3 (4.8)	10 (38.5)	**< 0.001**
Nicotine abuse, n (%)	22 (23.2)	0 (0)	14 (22.2)	8 (30.8)	0.261
Simple hernia, n (%)	62 (65.3)	4 (66.7)	45 (71.4)	13 (50)	0.158
Combined hernia, n (%)	33 (34.7)	2 (33.3)	18 (28.6)	13 (50)	
EHS hernia type, n (%)					
*M1*	2 (2.1)	0 (0)	1 (1.6)	1 (3.8)	0.163
*M2*	43 (45.3)	6 (100)	26 (41.3)	11 (42.3)	
*M3*	73 (76 − 8)	2 (33.3)	47 (74.6)	24 (92.3)	
*M4*	9 (9.5)	0 (0)	7 (11.1)	2 (7.7)	
*M5*	3 (3.2)	0 (0)	2 (3.2)	1 (3.8)	
Rectus diastasis, n (%)	19 (20)	1 (16.7)	13 (20.6)	5 (19.2)	0.967
Break gap [cm²], median (IQR)	6 (9.50)	3.1 (6.5)	6 (9.25)	6 (9.63)	0.254
Mesh size [cm²], median (IQR)	450 (150)	212.5 (300)	450 (120)	450 (131.25)	0.129
OP- time [min], median (IQR)	108 (61)	116 (39.75)	104 (65)	108 (60.75)	0.498

ASA - American Society of Anesthesiologists Physical Status Classification System

BMI - Body Mass Index [kg/m²]

EHS - Incisional Hernia Classification for ventral hernia of the European Hernia Society

SD - Standard deviation

IQR - Interquartile range

### ASA score

Age tended to increase with higher ASA scores, with significant differences observed between the ASA groups. Patients with an ASA score of I had a median age of 45.33 ± 11.60 years, those with a score of II had a median age of 49.51 ± 11.95 years, and those with a score of III had a median age of 60.23 ± 13.31 years. The median body mass index (BMI) of the overall patient cohort was 31 kg/m². A positive association between BMI and ASA score was observed. Patients with an ASA score of I had a median BMI of 25 kg/m² (IQR 21.75–31.75), those with a score of II had a median BMI of 30 kg/m² (IQR 27–35), and those with a score of III had a median BMI of 35.9 kg/m² (IQR 29.75–41.75). It is noteworthy that the majority of patients were non-smokers (approximately 77%). No significant association was found between smoking status and ASA score. The distribution of ASA scores within this patient cohort revealed that (approximately 66%, *n* = 63) were classified as ASA score II. This classification denotes patients with mild systemic disease but no functional limitations [35], including individuals who smoke or have a BMI between 30 and 40 kg/m². ASA score I is assigned to healthy, non-smoking individuals with minimal alcohol consumption, while ASA score III is given to patients with at least one severe systemic disease causing functional limitations, such as those with a BMI exceeding 40 kg/m².

When examining common comorbidities, cardiovascular disease was the most prevalent, affecting approximately 52% (*n* = 49) of the cohort. A significant association was observed between the presence of cardiovascular disease and higher ASA scores. Diabetes mellitus was present in 12.6% (*n* = 12) of patients, and 13.7% (*n* = 13) were on regular anticoagulation therapy. Of these conditions, only anticoagulation therapy showed a statistically significant relationship with ASA classification. Pulmonary disease was present in just over 2% (*n* = 2) of patients, with no significant association identified in relation to ASA classification.

### Complications

Intraoperative complications occurred in 2.1% (*n* = 2) of cases. Red blood cell concentrates were administered to 1% (*n* = 1) of patients, while no patients required postoperative antibiotic therapy. The overall postoperative complication rate was approximately 7.4% (*n* = 7). According to the Clavien-Dindo grading system, 2.1% (*n* = 2) of complications were graded as I, 1.1% (*n* = 1) as II, and 4.2% (*n* = 4) as III. Two complications were classified as Clavien-Dindo grade I: One case of subcutaneous hematoma and one case of hemoglobin-relevant secondary hemorrhage. A superficial postoperative hemorrhage managed at the bedside was categorized as Clavien-Dindo grade II. Four complications were assigned to Clavien-Dindo grade IIIb. These included surgical evacuation of a hematoma, reoperation on the day of surgery due to hemorrhage, reoperation for early recurrence, and a deep wound abscess requiring vacuum-assisted closure (VAC) therapy. There was no statistically significant difference in the distribution of Clavien-Dindo grades among ASA groups (*p* = 0.058). Regarding specific postoperative complications, surgical site occurrences (SSOs) were observed in 5.3% (*n* = 5) of cases, while surgical site infections (SSIs) occurred in 1.1% (*n* = 1). No cases of postoperative ileus and necessity of antibiotics were recorded. Hernia recurrence within the first year postoperatively was observed in 5.3% (*n* = 5) of patients. Intraoperative placement of drains to evacuate wound fluid was performed in 24.2% (*n* = 23) of cases. The median postoperative length of hospital stay was 3 days (IQR 2–3). None of these variables showed statistically significant associations with ASA score. Further details can be found in Table 2.

**Table 2:** Complications and further descriptions

	Total	ASA score I	ASA score II	ASA score III	*p*-value
Number of patients, n (% of the patient dataset)	**95 (100)**	**6 (6.3)**	**63 (66.3)**	**26 (27.4)**	
Intraoperative complications, n (%)	2 (2.1)	0 (0)	1 (1.6)	1 (3.8)	0.743
Drainage, n (%)	23 (24.2)	1 (16.7)	14 (22.2)	8 (30.8)	0.628
Min. 1 RBC, n (%)	1 (1.1)	0 (0)	0 (0)	1 (3.8)	0.262
Postoperative complications, n (%)	7 (7.4)	0 (0)	3 (4.8)	4 (15.4)	0.169
Clavien-Dindo 0-III, n (%)					
0	88 (92.6)	6 (100)	60 (95.2)	22 (84.6)	0.058
I	2 (2.1)	0 (0)	2 (3.2)	0 (0)	
II	1 (1.1)	0 (0)	0 (0)	1 (3.8)	
III	4 (4.2)	0 (0)	1 (1.6)	3 (11.5)	
SSO, n (%)	5 (5.3)	0 (0)	2 (3.2)	3 (11.5)	0.230
SSI, n (%)	1 (1.1)	0 (0)	0 (0)	1 (3.8)	0.262
Length of hospitalisation in days, median (IQR)	3 (1)	3 (0.5)	3 (1)	3 (1.25)	0.119
Recurrence within 1 year, n (%)	5 (5.3)	0 (0)	2 (3.2)	3 (11.5)	0.230

RBC - Red blood cell concentrate, erythrocyte concentrate

SSO - Surgical site occurrence

SSI - Surgical site infection

IQR - Interquartile range

### Logistic regression on ASA score and intraoperative complications

A binary logistic regression analysis was conducted to explore the potential association between ASA score classification and the occurrence of intraoperative complications. In this model, intraoperative complications were the dependent variable, while ASA score served as the independent variable. The omnibus test of model coefficients, Nagelkerke R², and Hosmer-Lemeshow test were used to evaluate the quality of the binary logistic regression model assessing the potential association between ASA score and intraoperative complications. The omnibus test indicated no statistical significance (x²(1) = 0.582, *p* = 0.446), suggesting that ASA score did not reliably predict the risk of intraoperative complications in this cohort. The model yielded a Nagelkerke R² of 0.033, reflecting a low proportion of explained variance. The Hosmer-Lemeshow test demonstrated good model fit (*p* = 0.844). The chi-square statistic of 0.039 (df = 1) further supports the lack of a statistically significant effect of the predictor on the outcome. The analysis did not demonstrate a statistically significant association between ASA score and intraoperative complications (*p* = 0.453, 95% CI [0.026, 5.101]), see Table 3.

**Table 3:** Results of univariate binomial logistic regression between intraoperative complications and ASA score

Logistic Regression - intraoperative complications and ASA score								
	B	SE	Wald	df	Sig.	Exp(B)	95% CI for EXP(B)	
							Lower Bound	Upper Bound
ASA score	−1.010	1.347	0.563	1	0.453	0.364	0.026	5.101
Constant	6.217	3.434	3.278	1	0.070	501.094		

B - Regression coefficient (logit coefficient)

SE - Standard Error

Wald - Wald test statistic

Df - Degrees of Freedom

Sig. - Significance level (p-value)

Exp (B) - Exponentiated B (Odds Ratio)

CI - Confidence Interval for Exponentiated B (Odds Ratio)

### Logistic regression on ASA score and postoperative complications

The relationship between ASA score and the occurrence of postoperative complications was further explored using binary logistic regression. The omnibus test of model coefficients, Nagelkerke R², and Hosmer-Lemeshow test were used to evaluate the quality of the binary logistic regression model assessing the potential association between ASA score and postoperative complications. The omnibus test did not reach statistical significance (x²(1) = 3.382, *p* = 0.066), suggesting that ASA classification alone does not significantly improve the prediction of postoperative complications compared to a null model. The model yielded a Nagelkerke R² of 0.085, indicating a limited proportion of variance explained. The Hosmer-Lemeshow test confirmed adequate model fit (*p* = 0.775). The chi-square statistic of 0.082 (df = 1) further indicates that the predictor variable did not have a statistically significant effect on the outcome. No statistically significant association was observed (*p* = 0.076, 95% CI [0.057, 1.155]). While not statistically significant, a trend towards increased complication rates was observed among patients with ASA score III (see Table 4).

**Table 4:** Results of univariate binomial logistic regression between postoperative complications and ASA score

Logistic Regression - postoperative complications and ASA score								
	B	SE	Wald	df	Sig.	Exp(B)	95% CI for EXP(B)	
							Lower Bound	Upper Bound
ASA score	−1.364	0.769	3.144	1	0.076	0.256	0.057	1.155
Constant	5.775	1.994	8.387	1	0.004	322.034		

B - Regression coefficient (logit coefficient)

SE - Standard Error

Wald - Wald test statistic

Df - Degrees of Freedom

Sig. - Significance level (p-value)

Exp (B) - Exponentiated B (Odds Ratio)

CI - Confidence Interval for Exponentiated B (Odds Ratio)

### Multivariate logistic regression - postoperative complications

In the multivariate logistic regression model with the variables age, gender, BMI, cardiovascular disease patients, lung disease patients, diabetes mellitus, intake of anticoagulation, nicotine abuse, break gap, mesh size and duration of surgery, a significant association was only found for mesh size (*p* = 0.049, 95% CI [1.000, 1.028]). The odds ratio of 1.014 indicates that for each unit increase in mesh size, the odds of the outcome increase by approximately 1.4% (see Table 5).

**Table 5:** Results of multivariate binomial logistic regression between postoperative complications and ASA score

Multivariate Logistic Regression for postoperative complications								
	B	SE	Wald	df	Sig.	Exp(B)	95% CI for EXP(B)	
							Lower Bound	Upper Bound
Age	0.000	0.057	0.000	1	0.994	1.000	0.895	1.118
Gender	−1.290	1.330	0.940	1	0.332	0.275	0.020	3.734
BMI	−0.117	0.089	1.711	1	0.191	0.890	0.030	8.115
Cardiovascular disease	0.884	1.492	0.352	1	0.553	2.422	0.130	45.050
Lung disease	5.153	3.067	2.822	1	0.093	172.883	0.424	70531.497
Diabetes mellitus	−17.913	10196.843	0.000	1	0.999	0.000	0.000	-
Anticoagulation	−0.196	1.832	0.011	1	0.915	0.822	0.023	29.785
Nicotine abuse	−1.212	1.938	0.391	1	0.532	0.298	0.007	13.276
Break gap	0.018	0.055	0.110	1	0.741	1.018	0.915	1.133
Mesh size	0.014	0.007	3.869	1	**0.049**	1.014	1.000	1.028
OP-time	−0.016	0.013	1.597	1	0.206	0.984	0.595	1.009

B - Regression coefficient (logit coefficient)

SE - Standard Error

Wald - Wald test statistic

Df - Degrees of Freedom

Sig. - Significance level (p-value)

Exp (B) - Exponentiated B (Odds Ratio)

CI - Confidence Interval for Exponentiated B (Odds Ratio)

## Discussion

In summary, the median length of hospitalization in our study was 3 days (IQR 2–3). A meta-analysis by Aliseda et al. (2022), which included 918 patients who underwent laparoscopic and robotic eTEP surgery, reported an average hospital stay of only 1.77 days, with an almost identical intraoperative complication rate of 2%, and a lower postoperative complications rate compared to our results [16]. This discrepancy may be attributed to differences in clinical conditions, patient cohorts, or surgical techniques.

The wound infection rate in our cohort was 1.1% (*n* = 1), compared to 0% in the aforementioned meta-analysis and furthermore studies [16, 36]. This difference may be explained by variations in study methodology. While Aliseda et al. (2022) examined a larger and more homogeneous patient population, our study involved a smaller cohort that may have had different risk factors. Furthermore, differences in postoperative management may also have contributed to the higher wound infection rate. Seromas and hematomas are a common postoperative complication, particularly in laparoscopic procedures. Based on published literature, their incidence ranges from 5% to 25% [37]. In our study, the seroma rate was 5.3% (*n* = 5), which is nearly identical to the 5% observed in the meta-analysis [16]. Mishra et al. (2022) also reported a similar seroma rate of 5.8% as a postoperative complication [38]. Higher rates of seromas, such as 10.3%, have occasionally been observed in smaller studies, such as that by Bellido Luque et al. (2021) [19, 39]. This suggests that seromas are a foreseeable complication in the context of eTEP surgery. Complications were graded using the Clavien-Dindo classification, a validated system widely adopted for reporting surgical adverse events based on the therapy required to manage them [40]. This allows for more meaningful comparison of complication severity across studies. According to the meta-analysis, serious complications classified as Clavien-Dindo grade III to IV occurred in approximately 1% of patients. In the present dataset, such complications were observed in 4.2% (*n* = 7) of cases, reflecting a slightly higher incidence. This discrepancy may be explained by differences in the patient dataset, a significantly smaller sample size in our study, and various factors such as differences in clinical practice, complication management protocols at each center, stricter inclusion and exclusion criteria, and variations in patient demographics. The 1-year recurrence rate in our cohort was 5.3% (*n* = 5). While this was not the primary focus of the present study, it is comparable to rates reported in larger eTEP series. For instance, Anusitviwat et al. (2025) reported a recurrence rate of 3.1% in their cohort, while Belyansky et al. (2021) documented a recurrence rate of 3.4% at 1 year in a multicenter study of robotic eTEP [17, 41]. Additionally, Kudsi et al. (2019) observed a recurrence rate of 4.1% with a mean follow-up of 18 months [42]. Our recurrence rate falls within the expected range (2–8%) for minimally invasive ventral hernia repair, though longer follow-up is needed to fully assess durability [7]. An important consideration is the distinction between retrospective and prospective data collection. In prospective studies, potential biases arising from selection or observation errors are minimized, allowing for a more accurate assessment of complication rates.

Our findings also show that patients with an ASA score of I experienced no complications, whereas complications occurred exclusively in patients with ASA scores of II and III. This suggests a potential association between higher ASA scores and increased perioperative risk. A large prospective study involving 6,301 patients across various surgical procedures reported a significant association between ASA classification and perioperative complications, with odds ratios of 2.2 for ASA III and 4.2 for ASA IV, indicating a progressively higher risk with increasing ASA scores [24]. However, in our cohort, ASA score was not significantly associated with either intraoperative or postoperative complications. In both univariate analyses, the p-values for ASA score were high, suggesting it was not a reliable predictor of complication risk in this sample. In contrast, multivariate binary logistic regression, which included other potential predictors, revealed a significant association between increasing mesh size and postoperative complications. No such association was observed for defect size. Although previous studies have reported associations between defect size and complications such as seroma, pain, or ileus, further research is warranted to clarify the role of mesh size in postoperative outcomes [43]. Several important statistical limitations warrant emphasis. The low event rates—while reflecting eTEP’s favorable safety profile—significantly limit the statistical power of our regression analyses. With only 2 intraoperative complications, any multivariate modeling is unreliable and these results should not be interpreted as definitive (see Appendix Table 6). For postoperative complications (*n* = 7), our univariate analysis of ASA score achieves an events-per-variable ratio of 7:1, which is adequate for exploratory analysis but below ideal thresholds for confirmatory inference. The multivariate model with 11 covariates (events-per-variable ratio of 0.64) is severely underpowered. Our logistic regression analyses should be viewed as hypothesis-generating, providing effect estimates and confidence intervals that may inform future adequately powered studies and meta-analyses, rather than definitive risk prediction models. Meaningful statistical inference regarding predictors of rare complications is limited under these conditions.

Another limitation of our study is the absence of robotic-assisted eTEP cases, which reflects the lack of an available robotic platform during the study period rather than a deliberate exclusion. Consequently, direct comparisons between conventional and robotic eTEP procedures could not be made.

One potential explanation for the lack of association between ASA score and complications may be the heterogeneous and highly imbalanced distribution of ASA classifications in our dataset. Only six patients were classified as ASA I, severely limiting statistical power and increasing the risk of Type II error. While the absence of complications among ASA I patients aligns with the general principle that healthier individuals carry lower perioperative risk, this observation should be interpreted with caution [8, 9]. The underrepresentation of ASA I patients, and the absence of ASA IV patients, prevents meaningful subgroup analysis. A larger, more balanced cohort might indeed reveal a significant association between ASA score and postoperative outcomes.

These findings also raise the question of whether the ASA score alone is a sufficiently reliable predictor of perioperative risk in clinical practice. The ASA classification aggregates a broad spectrum of comorbidities into discrete categories and may lack the granularity required to detect subtle associations with perioperative outcomes in this context. Although several studies have shown a correlation between ASA class and surgical outcomes, the classification system has well-documented limitations. It is subjective in nature and susceptible to interobserver variability, as scoring can differ based on the anesthesiologist’s interpretation and the patient’s reported history. Furthermore, the system does not account for procedure-specific risk factors, and risk may vary substantially within a given ASA category. Given the small and uneven distribution of ASA scores in our sample, particularly the limited number of low and high ASA class patients, our findings should not be overinterpreted. Future studies with larger and more evenly distributed ASA groups are needed to more accurately assess the predictive value of ASA classification in the context of perioperative risk.

All procedures were performed by a single, experienced surgeon. While this enhances internal consistency in technique and perioperative management, it inherently limits the generalizability of our findings. Outcomes, particularly complication rates and operative times, may vary in broader clinical practice across surgeons with different experience levels or technical approaches to eTEP. Multicenter studies involving multiple surgeons are essential to confirm the broader applicability of our observations regarding ASA score and risk.

This study is inherently limited by its retrospective, single-arm design. The absence of a control group (e.g., open repair or IPOM) prevents direct comparative effectiveness conclusions between techniques. However, our study design is consistent with the vast majority of published eTEP literature, which consists predominantly of single-center retrospective series. This foundational descriptive approach is a necessary precursor to the comparative trials and randomized controlled studies that represent the next phase of eTEP research. Our data provide benchmarking information and effect estimates that can inform power calculations for future comparative studies. Our analysis focused on the association between ASA score and complications within the eTEP cohort, a design employed in prior studies assessing ASA risk in specific procedures [25, 26]. However, the retrospective nature and the resulting limitations in controlling for all potential confounders must be acknowledged. Furthermore, the relatively small overall sample size (*n* = 95), coupled with the low incidence of complications and the marked imbalance in ASA group sizes (especially ASA I, *n* = 6), significantly reduced the statistical power to detect associations, likely contributing to the lack of statistical significance despite observed trends (e.g., higher complications in ASA III). This underscores the need for larger, prospective, potentially multi-institutional studies to achieve adequate power for robust risk stratification.

Future studies should seek to overcome these limitations by including larger multicenter cohorts and comparator groups involving open, laparoscopic, or robotic assisted repairs. Such designs would not only enhance statistical power but also permit a more refined analysis of the interplay between ASA classification and surgical technique. Prospective data collection, long term follow up, and integration of patient reported outcomes will be essential to validate and expand upon the exploratory findings presented here.

## Conclusion

In this cohort of patients undergoing eTEP ventral hernia repair, ASA score was not a statistically significant predictor of intraoperative or postoperative complications. However, the exclusive occurrence of complications in ASA II and III patients, alongside the observed trend towards higher complication rates in ASA III patients, suggests a potential clinical relevance of ASA status that warrants further investigation. The extreme imbalance in ASA group distribution, particularly the very small ASA I subgroup, limited the statistical power to draw definitive conclusions. Our data question the utility of the ASA classification as a standalone predictive tool for eTEP-specific complications and underscore the importance of developing more nuanced, procedure-specific risk assessment models. Further prospective, multicenter studies with larger, more balanced cohorts are warranted to clarify the role of ASA score and identify reliable predictors for risk stratification in eTEP procedures.

## Data Availability

The data presented in this study are available on reasonable request from the corresponding author. The data are not publicly available due to privacy and ethical restrictions.

## Appendix

**Table 6:** Results of multivariate binomial logistic regression - intraoperative complications

Multivariate Logistic Regression for intraoperative complications								
	B	SE	Wald	df	Sig.	Exp(B)	95% CI for EXP(B)	
							Lower Bound	Upper Bound
Age	-1.496	5350.321	0.000	1	1.000	0.224	0.000	-
Gender	24.000	325987.126	0.000	1	1.000	26479684041	0.000	-
BMI	-3.576	2948.930	0.000	1	0.999	0.028	0.000	-
Cardiovascular disease	-0.935	119626.245	0.000	1	1.000	0.392	0.000	-
Lung disease	-3.573	166350.727	0.000	1	1.000	0.028	0.000	-
Diabetes mellitus	1.047	57233.582	0.000	1	1.000	2.850	0.000	-
Anticoagulation	86.893	275356.971	0.000	1	1.000	5.458E + 37	0.000	-
Nicotine abuse	57.069	70228.238	0.000	1	0.999	6.094E + 24	0.000	-
Break gap	1.664	5639.027	0.000	1	1.000	5.281	0.000	-
Mesh size	0.016	499.543	0.000	1	1.000	1.016	0.000	-
OP-time	0.471	505.225	0.000	1	0.999	0.624	0.000	-

B - Regression coefficient (logit coefficient)

SE - Standard Error

Wald - Wald test statistic

Df - Degrees of Freedom

Sig. - Significance level (p-value)

Exp (B) - Exponentiated B (Odds Ratio)

CI - Confidence Interval for Exponentiated B (Odds Ratio)

## Abbreviations

eTEP: Extended View Totally Extraperitoneal Repair
ASA: American Society of Anesthesiologists Physical Status Classification System
SSO: Surgical site occurrence
SSI: Surgical site infection
RBC: Red blood cell concentrate, erythrocyte concentrate
EHS Classification: Incisional Hernia Classification of the European Hernia Society
VHRI: Ventral Hernia Recurrence Inventory
